# The elephant shark methylome reveals conservation of epigenetic regulation across jawed vertebrates

**DOI:** 10.12688/f1000research.11281.1

**Published:** 2017-04-20

**Authors:** Julian R. Peat, Oscar Ortega-Recalde, Olga Kardailsky, Timothy A. Hore

**Affiliations:** 1 Department of Anatomy, University of Otago, Dunedin, 9016, New Zealand

**Keywords:** DNA methylation, epigenetics, elephant shark, vertebrate, evolution, cartilaginous fish, gene regulation

## Abstract

Background: Methylation of CG dinucleotides constitutes a critical system of epigenetic memory in bony vertebrates, where it modulates gene expression and suppresses transposon activity. The genomes of studied vertebrates are pervasively hypermethylated, with the exception of regulatory elements such as transcription start sites (TSSs), where the presence of methylation is associated with gene silencing. This system is not found in the sparsely methylated genomes of invertebrates, and establishing how it arose during early vertebrate evolution is impeded by a paucity of epigenetic data from basal vertebrates.

Methods: We perform whole-genome bisulfite sequencing to generate the first genome-wide methylation profiles of a cartilaginous fish, the elephant shark *Callorhinchus milii*. Employing these to determine the elephant shark methylome structure and its relationship with expression, we compare this with higher vertebrates and an invertebrate chordate using published methylation and transcriptome data.

Results: Like higher vertebrates, the majority of elephant shark CG sites are highly methylated, and methylation is abundant across the genome rather than patterned in the mosaic configuration of invertebrates. This global hypermethylation includes transposable elements and the bodies of genes at all expression levels. Significantly, we document an inverse relationship between TSS methylation and expression in the elephant shark, supporting the presence of the repressive regulatory architecture shared by higher vertebrates.

Conclusions: Our demonstration that methylation patterns in a cartilaginous fish are characteristic of higher vertebrates imply the conservation of this epigenetic modification system across jawed vertebrates separated by 465 million years of evolution. In addition, these findings position the elephant shark as a valuable model to explore the evolutionary history and function of vertebrate methylation.

## Introduction

The methylation of DNA at cytosine bases constitutes an epigenetic regulatory system that is essential for the development of bony vertebrates ^1– 3^. Of particular significance is the modification of CG dinucleotides, whose symmetry allows methylation signals in this context to be perpetuated by maintenance methyltransferases following DNA replication ^4^. CG methylation and the epigenetic memory encoded by it thus form a stable but flexible storage system for molecular information.

The methylomes of studied vertebrates – including bony fish, amphibians and mammals – exhibit similar global patterns in which the majority of CG sites are methylated in somatic tissues ^5– 9^. Regulatory elements such as promoters and enhancers are an important exception to this pervasive methylation landscape, particularly when associated with short CG-rich regions termed CpG islands. At the transcription start site (TSS), the presence of methylation is associated with transcriptional silencing, an effect achieved through the inhibition of transcription factor binding and the action of proteins that recognise methylated DNA and induce an inaccessible chromatin configuration ^10, 11^. The inverse relationship of TSS methylation with gene expression has been documented across a wide range of vertebrate taxa ^5, 8, 12– 16^, indicating an evolutionarily important function. The molecular machinery that invokes an inactive state in response to methylation signals also appears to be conserved ^10^. Differences in methylation at regulatory regions are linked to the definition of cell fate during developmental progression and the stable maintenance of this identity in differentiated tissues ^5, 17– 19^. Indeed, widespread erasure of methylation marks in the cells of humans and mice plays a prominent role in the reprogramming of fate specification in both natural and experimental systems ^17, 20, 21^.

High levels of methylation outside the TSS of genes also serve an important function in vertebrate genomes. A substantial fraction of vertebrate genomes is composed of repetitive transposable elements (TEs), whose activity must be repressed to safeguard genome integrity ^22, 23^. These elements are ubiquitously methylated in vertebrate somatic tissues ^8, 9, 16, 24^, and experiments performed in mammalian model systems has shown this to be critical for their transcriptional repression ^25^. Hypermethylation of gene bodies is also a conserved feature of vertebrate genomes, and – unlike methylation at the TSS – this is compatible with active transcription in all species profiled to date ^5, 8, 12, 14– 16, 26– 28^. Although the relationship of intragenic methylation with gene expression levels is complex and appears to vary across taxa and even cell-type ^5, 8, 12– 16, 26, 28, 29^, it has been shown to suppress spurious transcription ^30^ and regulate exon splicing ^31, 32^ in mammalian systems.

The distribution and regulatory functions of methylation in vertebrates are unique amongst the metazoa, but the evolution of this system is poorly understood. In striking contrast to the pervasive hypermethylation that characterises vertebrates, invertebrate genomes are sparsely methylated and certain species such as the nematode *Caenorhabditis elegans* and fruit fly *Drosophila melanogaster* are apparently devoid of cytosine methylation ^14, 33– 36^. Where present, the predominant pattern is a mosaic configuration, in which unmethylated regions are interspersed with hypermethylated sequences, the latter preferentially located in gene bodies and in loose positive association with transcription ^14, 33– 35, 37^. Significantly, invertebrates lack the inverse relationship between TSS methylation and expression that constitutes a key regulatory mechanism in vertebrates, and the low levels of methylation do not appear to act as a control against TE activity in their genomes ^14, 35, 38– 41^.

**Figure 1.**
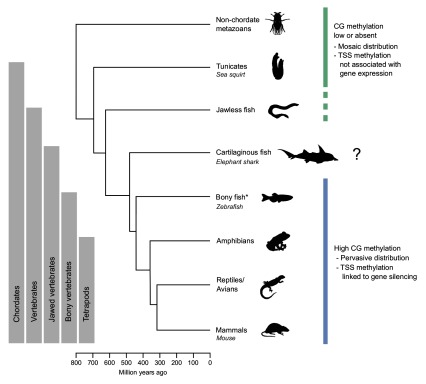
Phylogenetic tree showing major vertebrate groups, invertebrate outgroups and defining characteristics of their methylomes. The genomes of higher vertebrates are pervasively hypermethylated, with the exception of regulatory elements such as transcription start sites (TSSs), where the presence of methylation is associated with gene silencing (blue line). In contrast, invertebrate genomes are generally sparsely methylated in a mosaic pattern, and lack the inverse relationship between TSS methylation and expression that characterises vertebrates (green line). Certain invertebrate species appear to lack methylation altogether. Due to a paucity of data from basal vertebrate species, the evolutionary history of the CG methylation system present in higher vertebrates is unclear. Preprint methylation data from the sea lamprey *Petromyzon marinus* is not indicated here (see discussion). The names of organisms examined in this study are noted underneath the appropriate class. * The lobe-finned fish (sarcopterygii), as well as the cephalochordata (a basal chordate taxon), have been omitted for clarity. The following terms have been treated as equivalent: jawless fish and cyclostomata, jawed vertebrates and gnathostomata, cartilaginous fish and chondrichthyes, bony vertebrates and euteleostomi. Median divergence times from the TimeTree database ^43^ were used to construct the tree.

Methylation in *Ciona intestinalis*, a sea squirt belonging to the subphylum tunicata, the chordate lineage most closely related to vertebrates ^42^ ( Figure 1), typifies the invertebrate mosaic pattern ^14, 33, 35^. The methylation system present in higher vertebrates can thus be inferred to have evolved at some point after the divergence of tunicates from vertebrate progenitors (~680 Mya ^43^) and before the radiation of bony fish and tetrapods (~430 Mya ^43^; Figure 1). Understanding the timing of this progression at greater resolution and the factors that stimulated its development is hindered by the absence of methylation data from basal vertebrate classes.

Here, we use whole-genome bisulfite sequencing to generate the first methylation profiles of a cartilaginous fish, the elephant shark *Callorhinchus milii*. Through detailed comparison with published methylation and expression datasets, we demonstrate that the elephant shark methylome is characteristic of vertebrates in its global hypermethylation – including at TEs and gene bodies – and, crucially, association with transcriptional silencing at the TSS. These findings indicate conservation of a complex methylation system across jawed vertebrates separated by 465 million years of evolution, and identify the elephant shark as an important model to examine the origins and function of methylation in vertebrates.

## Methods

### Tissue source and DNA extraction

Elephant shark tissue samples were sourced as by-product of deceased animals harvested from commercial fishing in the Otago coastal region. As such, no animal ethics permission was applicable in this circumstance. No animal experimentation or manipulation was undertaken as defined by the Animal Welfare Act (2009, New Zealand), or according to guidelines issued by the New Zealand National Animal Ethics Advisory Committee (NAEAC, Occasional Paper No 2, 2009, ISBN 978-0-478-33858-4).

DNA was purified using a modified magnetic bead approach ^44^. Briefly, cells were first homogenised in “GITC” lysis buffer (4 M Guanidine thiocyanate, Sigma G6639; 50 mM Tris, Thermo 15568-025; 20 mM EDTA; Thermo 15575-020; 2% Sarkosyl, Sigma L9150-50G; 0.1% Antifoam, Sigma A8311-50ML), and this lysate mixture was then combined with TE-diluted Sera-Mag Magnetic SpeedBeads (GE Healthcare, GEHE45152105050250) and isopropanol in a volumetric ratio of 2:3:4, respectively. Following capture with a neodymium magnet, beads were washed once with isopropanol, twice with 70% ethanol and resuspended in filter-sterile milliQ water.

### Preparation of WGBS-seq libraries

WGBS-seq was undertaken using a post-bisulfite adapter tagging (PBAT) method adapted from Peat *et al.*, 2014 ^45^. Briefly, 50–100 ng of purified DNA was subjected to bisulfite conversion using the Imprint DNA modification kit (Sigma, MOD50). Converted DNA underwent first strand synthesis with a biotin-labelled adapter sequence possessing seven random nucleotides at its 3’ end (BioP5N7, biotin- ACACTCTTTCCCTACACGACGCTCTTCCGATCTNNNNNNN). The product of first strand synthesis was captured using streptavidin-coated Dynabeads (Thermo, 11205D) and magnetic immobilisation. Double-stranded DNA was created using the immobilized first-strand as a template and an additional adapter that also possesses seven random nucleotides at its 3’ end (P7N7, GTGACTGGAGTTCAGACGTGTGCTCTTCCGATCTNNNNNNN). Unique molecular barcodes and sequences necessary for binding to Illumina flow-cells were added to libraries by PCR using 1X HiFi HotStart Uracil+ Mix (KAPA, KK2801 and 10 μM indexed Truseq-type oligos), with thermal cycling as follows: 12× (94°C, 80 sec; 65°C, 30 sec; 72°C, 30 sec).

For deep sequencing, libraries were sequenced with a single-end 100bp protocol on a HiSeq 2500 instrument (Illumina) using rapid run mode. For low-coverage sequencing of additional samples, libraries were sequenced on a MiSeq instrument (Illumina) until the desired depth (at least 15,000 mapped CG calls) was attained.

Detailed sequencing results are provided in Table S1. 

### Bioinformatic processing of WGBS-seq dataset

Mapped CG methylated calls for mouse liver ^5^ were downloaded from GEO (accession GSE42836, sample GSM1051157) and analysed directly. For zebrafish muscle ^8^ ( SRA study SRP020008, run SRR800081) and sea squirt muscle ^14^ (GEO accession GSE19824, sample GSM497251), raw sequencing data was downloaded and processed along with elephant shark WGBS-seq data generated in this study as follows.

Trimming was performed to remove both poor-quality calls and adapters sequences using TrimGalore (v0.4.0, default parameters). For the elephant shark data, 10bp were also removed from the 5’ end of reads to account for sequence biases associated with PBAT library construction.

Trimmed reads were aligned using Bismark ^46^ (v0.14.3, default parameters) with the --pbat option specified for elephant shark data. The following genome assemblies were used for alignment: zebrafish, GRCz10; elephant shark, 6.1.3; sea squirt, KH. For sea squirt and elephant shark, alignment was only performed against scaffolds larger than 277kb to avoid gene annotation issues and assembly artefacts. The deep-sequenced elephant shark data generated in this study was additionally mapped to the mitochondrial genome.

Bismark mapping reports were used to determine global methylation levels for low-coverage elephant shark data. All other datasets were deduplicated and CG methylation calls extracted using Bismark (--comprehensive and --merge_non_CG options specified). 

The number of mapped cytosine calls for sequencing performed in this study are provided in Table S1. The frequency of non-CG methylation indicates the maximum rate of non-conversion during the bisulfite treatment step; by this measure, all libraries had a bisulfite conversion efficiency of at least 98.9%.

### Bootstrap sampling to determine margin of error in low-coverage WGBS-seq

In order to determine the number of CG methylation calls required to accurately predict genome-wide methylation levels, bootstrap sampling of reads from the deep-sequenced male elephant shark dataset was performed to generate regular intervals of CG calls from approximately 100 to 30,000. These reads were trimmed, mapped and methylation quantified as described above, and following 1000 iterations, the proportion of data falling within the 0.5-99.5 percentiles was calculated to generate a 99% confidence interval. An asymptotic model described by the equation $y=2.208/\sqrt{x}$ was used to fit a curve to the data. At our minimum sequencing depth of 15,000 CG calls, bootstrap sampling predicts a margin of error (99% confidence interval) of approximately ±1.8 methylation percentage points.

### Analysis of deep-sequenced WGBS-seq datasets

CG methylation calls were imported into the SeqMonk program (v1.37.1) for analysis. For elephant shark and sea squirt, custom SeqMonk genomes were built using GFF annotation files downloaded from NCBI and Ensembl, respectively.

To analyse methylation at the level of individual CG dinucleotides, we generated an annotation track of each CG site using Bowtie v1.1.2 ^47^. A minimum of five methylation calls was required for inclusion of a CG site in analyses.

For mouse, zebrafish and elephant shark, precompiled annotation tracks of repetitive elements generated using the RepeatMasker program were downloaded from UCSC. For sea squirt, we generated these annotations by running the RepeatMasker program (v4.0.6) on the KH assembly with the -s option and specifying *Ciona intestinalis* as the species. The various classes of transposable elements were extracted from these annotation files and where indicated, merged for analysis. A minimum of five calls was applied as a threshold for inclusion when quantifying individual elements.

To examine methylation profiles across genes or TEs and neighbouring sequences, methylation was quantified at individual CGs and the mean plotted across a size-standardised gene or TE as well as 10kb upstream and downstream regions, using the quantitation trend plot function. Figures were produced using Prism (GraphPad, v7), with smoothing applied to flanking regions by averaging 100 neighbours. 

Transcription start sites were defined as 200bp centred on the first nucleotide of an annotated mRNA, and a minimum of five methylation calls was applied as a threshold for inclusion in analyses. For analysis of gene bodies, 2kb running windows were quantified (with a minimum of 50 methylation calls applied for inclusion) within annotated mRNAs, excluding 1kb at the 5’ end, and the mean was reported for each mRNA.

Violin plots and histograms were drawn using the ggplot2 package ^48^ in R.

### Bioinformatic processing of RNA-seq datasets

We downloaded raw sequencing data from previous studies as follows; sea squirt muscle ^14^, GEO accession GSE19824, sample GSM497252; elephant shark liver ^49^, SRA study SRP013772, run SRR513760; zebrafish muscle ^8^, SRA study SRP020008, run SRR800045; mouse liver (ENCODE Consortium ^50, 51^), GEO accession GSE78583, sample GSM2072415.

Trimming was performed to remove both poor-quality calls and adapters sequences using TrimGalore (v0.4.0, default parameters). In addition, 12bp were removed from the 5’ end of sea squirt reads and 10bp from the 5’ end of both elephant shark and mouse reads to avoid sequence biases.

Trimmed reads were aligned to the reference genomes described above with HISAT2 ^52^ (v2.0.5) using single-end or paired-end mode, as appropriate. Known splice sites were specified from a file built from GTF annotation files downloaded from Ensembl (release 87) using the HISAT2 python script. No GTF file was available for elephant shark, so a GFF annotation file downloaded from NCBI was first converted to GTF format using the gffread program ( https://github.com/gpertea/gffread).

### Analysis of RNA-seq datasets

Alignments from HISAT2 were imported into the SeqMonk program, specifying a minimum mapping quality of 60 to select only uniquely aligned reads.

The RNA-seq quantitation pipeline was used to generate raw read counts across the exons of nuclear protein-coding genes with a correction for any DNA contamination. Counts were corrected by transcript length and genes were divided into quintiles according to expression level.

## Results

### Genome-wide methylation profiles of the elephant shark, *Callorhinchus milii*

To generate genome-wide methylation profiles, we extracted DNA from the liver tissue of one female and one male adult elephant shark and performed whole-genome bisulfite sequencing (WGBS-seq). Detailed sequencing results are provided in Table S1.

**Figure 2.**
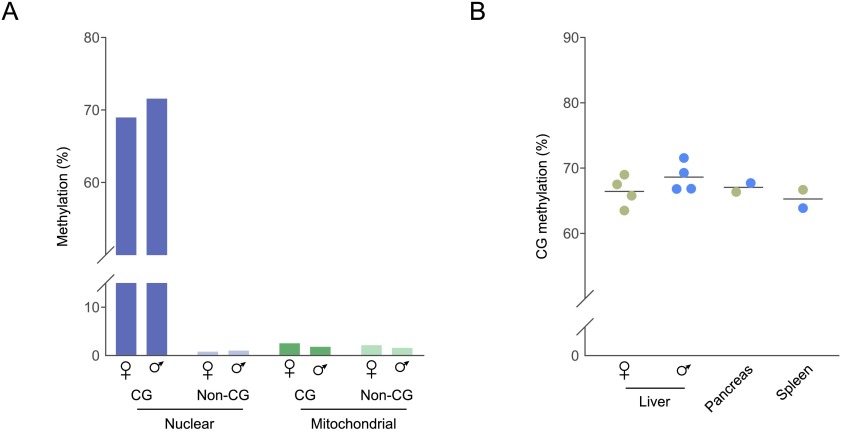
Global methylation levels in elephant shark somatic tissues. **A:** Global methylation levels of deep-sequenced liver samples in different contexts. ‘CG’ refers to symmetrical CG dinucleotides; ‘Non-CG’ indicates all other sequence contexts. **B:** Global CG methylation levels in elephant shark tissues examined by low-coverage sequencing. The horizontal bar indicates the mean; gold dots, female samples; blue dots, male samples. The difference between female and male liver samples is not significant according to a *t*-test, and within the technical margin of error expected at the threshold sequencing depth used (±1.8 methylation percentage points; Figure S1).

As described in the somatic tissues of other vertebrates, we found that methylation is much more prevalent in nuclear DNA at CG dinucleotides (69 – 71.6%) than in non-CG context (0.8 – 1%) or mitochondrial DNA (1.6 – 2.5%; Figure 2A). Low-coverage WGBS-seq demonstrated similar global methylation levels in three additional individuals for liver, and in spleen and pancreas samples ( Figure 2B). While we observed a small trend for lower methylation in female samples ( Figure 2B; female mean 66.4%, male mean 68.6%), this was not significant according to a *t*-test (p=0.2308) and within the margin of error expected at this sequencing depth ( Figure S1).

We proceeded with further analysis of CG methylation in deep-sequenced liver datasets as an example of the elephant shark somatic methylome, and combined male and female samples to enhance sequencing coverage.

### The elephant shark genome is pervasively methylated

Existing data indicate that methylation patterns differ markedly between vertebrates and invertebrates. In order to delineate the characteristics of these disparate systems and establish their relationship to the elephant shark methylome, we reanalysed published WGBS-seq data from two vertebrates, mouse ( *Mus musculus*) ^5^ and zebrafish ( *Danio rerio*) ^8^, as well as an invertebrate from the closest chordate outgroup, the sea squirt *Ciona intestinalis* ^14^ ( Table 1A).

**Table 1.** Published WGBS-seq and RNA-seq datasets used for comparative analysis in this study. Accession numbers are provided in the methods.

	A. WGBS-seq Datasets		B. RNA-seq Datasets	
Species	Reference	Tissue	Reference	Tissue
*Callorhinchus milii* Elephant shark	This study	Liver	Venkatesh *et al.*, 2014 ^49^	Liver
*Ciona intestinalis* Sea squirt	Zemach *et al.*, 2010 ^14^	Muscle	Zemach *et al.*, 2010 ^14^	Muscle
*Danio rerio* Zebrafish	Potok *et al.*, 2013 ^8^	Muscle	Potok *et al.*, 2013 ^8^	Muscle
*Mus musculus* Mouse	Hon *et al.*, 2013 ^5^	Liver	ENCODE Consortium ^50, 51^	Liver

**Figure 3.**
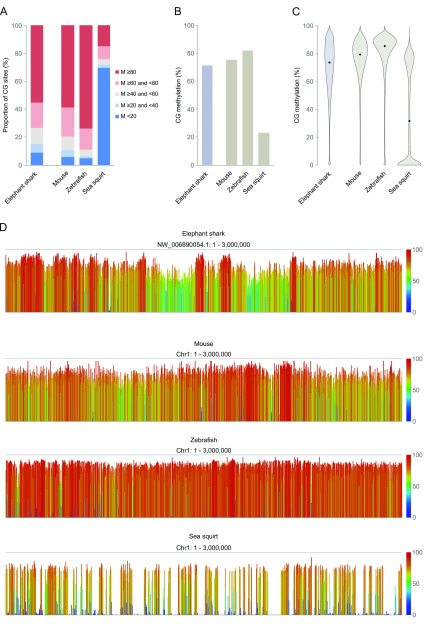
Global structure of the elephant shark methylome. **A:** Distribution of methylation at individual CG dinucleotides. ‘M’ denotes percentage CG methylation. **B:** Mean methylation of CG dinucleotides. **C:** Distribution of methylation within 2kb running windows covering the entire genome. Black dots denote the median. **D:** Genome screenshots of methylation quantified in 2kb running windows over the first 3Mb of chromosome 1 in sea squirt, zebrafish and mouse, and of the largest scaffold (NW_006890054.1) in elephant shark. These regions were arbitrarily chosen as an unbiased section of each genome.

As expected from analysis of global levels, examination of methylation at individual CG dinucleotides in the elephant shark showed that the majority of sites are highly methylated (≥ 80%), and fewer than one tenth are unmethylated ( Figure 3A). Both this pattern and the global methylation level are comparable to mouse and zebrafish ( Figure 3A–B). In contrast, mean methylation in the invertebrate sea squirt is only 22.9%, and over two thirds of CG sites are unmethylated.

A further striking distinction is evident when the genome is profiled in 2kb running windows. High methylation levels are pervasive in the elephant shark genome ( Figure 3C–D), resembling the structure of other vertebrate methylomes. In contrast, the sea squirt methylome is characterised by a bimodal but largely unmethylated distribution ( Figure 3C), resulting from a mosaic pattern in which background hypomethylation is punctuated by shorter stretches of methylated sequences ( Figure 3D). Interestingly, running windows show a broader distribution of methylation in elephant shark than in mouse or zebrafish ( Figure 3C). Whether this is a feature of basal vertebrates generally or of elephant shark specifically will require analysis of methylation patterns in additional cartilaginous fish.

### Transposable elements are hypermethylated in the elephant shark

Having established that the global structure of the elephant shark methylome is characteristic of vertebrates, we sought to determine the profile and impact of methylation at specific functional elements.

Transposable elements (TEs) are highly methylated in vertebrate genomes, a feature which is linked to the necessity of repressing their transcription to prevent destabilising transposase activity ^8, 9, 16, 22– 24^. The generally low levels of methylation at TEs in invertebrates such as the sea squirt do not appear to regulate their activity ^14, 38, 39^.

**Figure 4.**
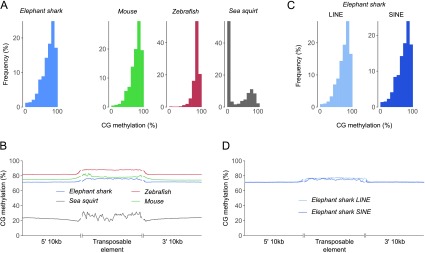
Methylation patterns at transposable elements. **A:** Distribution of methylation at transposable elements. Mean methylation values are divided into 10 bins. **B:** Mean CG methylation across transposable elements and 10kb flanking regions. Quantification was performed at the level of individual CG dinucleotides. Flanking regions were smoothed by averaging 100 neighbours. **C:** Distribution of methylation at long interspersed nuclear elements (LINEs) and short interspersed nuclear elements (SINEs) in the elephant shark genome. Mean methylation values are divided into 10 bins. **D:** Methylation at long interspersed nuclear elements (LINEs) and short interspersed nuclear elements (SINEs) in the elephant shark genome, plotted as in ( **B**).

Examination of methylation patterns at TEs and flanking sequences showed that the elephant shark exhibits hypermethylation at the large majority of TEs and a slight increase in mean methylation relative to adjacent regions ( Figure 4A–B), conforming to the pattern of other vertebrates. While mean methylation levels of TEs in sea squirt are moderately elevated compared to flanking sequences, the large majority of TEs are hypomethylated. Little variation in methylation was observed between the two predominant TE classes in the elephant shark genome, long interspersed nuclear elements (LINEs) and short interspersed nuclear elements (SINEs; Figure 4C–D), indicating that – as in other vertebrates ^8, 9, 16, 24^ – hypermethylation of TEs is ubiquitous. 

### Methylation at elephant shark transcription start sites is associated with gene silencing

Silencing of gene expression through the deposition of methylation at transcription start sites (TSSs) constitutes an important regulatory mechanism in vertebrates, but appears to be absent from invertebrates ^5– 10, 12– 16, 35, 40, 41^. To compare the relationship of methylation and transcription in elephant shark with higher vertebrates and the sea squirt, we made use of tissue-matched published RNA-seq datasets ^8, 14, 49, 50^ ( Table 1B) to classify protein-coding genes into expression quintiles.

**Figure 5.**
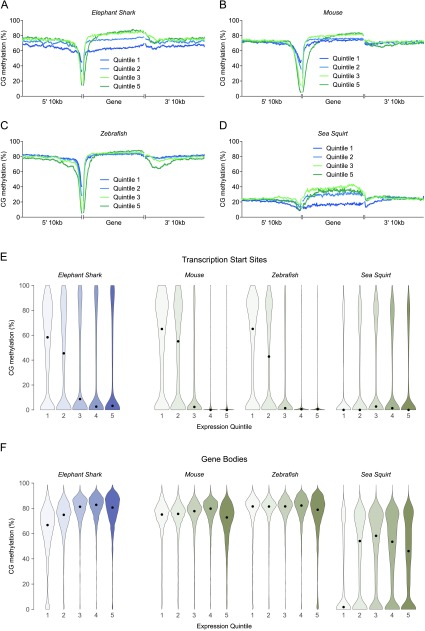
Relationship between methylation and gene expression. **A** – **D:** Mean CG methylation across genes and 10kb flanking regions, classified into quintiles according to expression level in RNA-seq datasets (5 = highest). Quintile 4 is omitted for clarity. Quantification was performed at the level of individual CG dinucleotides. Flanking regions were smoothed by averaging 100 neighbours. **E** – **F:** Distribution of methylation at the transcription start site ( **E**) and within the body ( **F**) of genes classified into quintiles according expression level (5 = highest). Each violin is scaled to the same maximum width (total area is not constant between violins) to demonstrate distributions for each quintile. Black dots denote the median.

Hypomethylation at the TSS of expressed genes constitutes a conspicuous exception to the otherwise pervasively methylated elephant shark genome, matching the higher vertebrates examined ( Figure 5A–C). Significantly, we document an inverse relationship between TSS methylation and expression level in the elephant shark ( Figure 5A, Figure 5E). A bimodal distribution in which a large proportion of sequences are methylated at low expression levels contrasts with negligible methylation at most TSSs of intermediate and highly expressed genes. The association of TSS methylation with transcriptional silencing is a distinguishing feature of higher vertebrate methylomes ^5, 8, 12– 16^ that is recapitulated here for zebrafish and mouse ( Figure 5B–C, Figure 5E), and its presence in the elephant shark indicates that methylation at the TSS induces repression in a similar manner. Consistent with reports showing that invertebrates lack this wide variation in TSS methylation as a function of expression level ^14, 35, 41^, the large majority of sea squirt TSSs are hypomethylated at all expression levels and methylation levels at the TSS are comparable to intergenic sequences ( Figure 5D–E).

Interestingly, a larger number of TSSs at highly expressed genes remain methylated in elephant shark compared to mouse and zebrafish. This may suggest that the association of methylation with repression is less absolute than in higher vertebrates, but could also be attributed to poorer TSS annotation in the less intensively-studied and incompletely assembled elephant shark genome.

The methylomes of higher vertebrates and invertebrates also differ within gene bodies. While intragenic methylation in sea squirt forms the bimodal distribution reported in invertebrates ^35, 37^, and most silenced genes lack methylation, vertebrate gene bodies are generally hypermethylated at all expression levels ( Figure 5F). Intragenic methylation in the elephant shark is characteristic of this vertebrate pattern. In addition, higher expression levels are associated with moderately elevated gene body methylation in elephant shark liver, but not in zebrafish muscle or mouse liver. Given the limited understanding of the role played by intragenic methylation in the regulation of vertebrate gene expression, the functional relevance of this relationship is unclear.

## Discussion

Methylation of CG dinucleotides forms a heritable but flexible epigenetic memory that constitutes a critical regulatory system in bony vertebrates, where it is employed in the modulation of gene expression and suppression of transposon element activity. The genomes of studied vertebrates are pervasively hypermethylated, with the exception of regulatory elements such as transcriptional start sites (TSSs), where the presence of methylation is linked to transcriptional silencing ^1– 10, 12– 16, 22– 25^. These features are not found in the sparsely methylated genomes of invertebrates, including chordates closely related to vertebrates ^14, 33– 40^, but establishing when this important regulatory system arose and the factors that drove its development has been has been impeded by a lack of methylation data from basal vertebrates ( Figure 1).

In this study, we employ WGBS-seq to generate the first genome-wide methylation profiles of a cartilaginous fish, the elephant shark *Callorhinchus milii*. Through detailed comparison with published methylation and expression datasets, we demonstrate that the elephant shark methylome is characteristic of higher vertebrates and in clear contrast to the prevailing invertebrate configuration. 

We first note that methylation in the elephant shark is primarily located in symmetric CG context, where comparable global methylation levels of approximately 65–70% were found by low-coverage WGBS-seq in the male and female liver, as well as in the spleen and pancreas ( Figure 2). The similarity of male and female methylation indicates that, unlike certain bony fish species ^53^, the uncharacterised sex-determination mechanism in the elephant shark is not associated with large differences in global methylation. Examination of liver profiles at higher resolution demonstrated that – like higher vertebrates – the majority of elephant shark CG sites are methylated, and this is ubiquitous throughout the genome rather than concentrated in short stretches in the invertebrate mosaic pattern, typified by the sea squirt ( Figure 3). The global hypermethylation of the elephant shark genome includes both major transposon classes, LINEs and SINEs ( Figure 4), whose transcriptional repression is thought to be an important function of vertebrate methylation systems as a safeguard against destabilising transposition activity.

Crucially, the elephant shark mirrors higher vertebrates in their inverse relationship of methylation with expression at the TSS ( Figure 5); most expressed genes are unmethylated while a large proportion of inactive genes are hypermethylated at the TSS. This indicates that TSS methylation represses gene expression in a similar fashion in the elephant shark, and implies that this key regulatory mechanism – which is absent from invertebrates – is present in cartilaginous fish. While the association of TSS methylation with silencing is conserved across the vertebrates examined, we also observe that a greater number of expressed genes are methylated at the TSS in elephant shark than in mouse or zebrafish. It will be important to clarify whether this arises from the poorer annotation of the less intensively studied elephant shark genome, or a meaningful biological difference in the repressive potency of methylation in this system.

The hypermethylation of most gene bodies at all levels of transcription is a feature of higher vertebrate methylomes, that our data show is also shared by the elephant shark ( Figure 5). We additionally document an interesting association between higher expression levels and elevated methylation in the elephant shark, a trend which is absent from the higher vertebrate tissues we examined. The relationship between intragenic methylation and expression is complex and appears to vary between vertebrate taxa and even within the tissues of a single species ^5, 8, 12– 16, 26, 28, 29^. Indeed, although a variety of functions for intragenic methylation have been suggested, including suppression of spurious transcription and regulation of exon splicing ^30– 32^, their generality is poorly understood, particularly outside mammalian systems. Significant further research will be required to uncover the impact of intragenic methylation in vertebrate genomes and determine the biological relevance of its positive relationship with expression in the elephant shark. 

### Evolutionary history of the vertebrate methylation system

The observation that methylation patterns in a cartilaginous fish are characteristic of higher vertebrates implies the conservation of a complex methylation system across jawed vertebrates separated by 465 million years of evolution ( Figure 1). Of particular note, they support the common presence of a regulatory architecture that links methylation at the TSS to transcriptional repression.

Preprint methylome data from the sea lamprey *Petromyzon marinus*, a basal jawless vertebrate, indicate that this species lacks the genome-wide hypermethylation and functional relationships of higher vertebrates ( https://doi.org/10.1101/033233). While the data from this study has not yet been released, the authors state that methylation patterns in sea lamprey more closely resemble those of the sea squirt and appear to represent a transitional intermediate. In the context of our findings, this implies that the evolution of the higher vertebrate methylation system was achieved after the emergence of jawed vertebrates (~600 Mya ^43^), but before the divergence of bony and cartilaginous fish (~465 Mya ^43^; Figure 1). These data further identify cartilaginous fish as the most divergent class to possess a DNA modification system similar to our own, and position the elephant shark as a valuable model to examine the function and evolution of the vertebrate methylation system. As the slowest evolving vertebrate documented ^49^, the elephant shark bears the closest resemblance to the most recent common ancestor of all jawed vertebrates, enhancing its appeal in this respect. Moreover, the extensive orthology of its small genome to those of tetrapods ^49^ facilitates comparative studies.

Transposon aggressiveness correlates with the degree of sexual outcrossing in the host, and repression of this destabilising activity has been proposed as a major reason for genome-wide hypermethylation in sexually-reproducing organisms such as plants and vertebrates ^14, 38, 54^. This control mechanism appears to have been discarded as unnecessary in early asexual metazoans, and alternative suppression systems such as the piwi-piRNA pathway were developed in their sexually-reproducing invertebrate descendants ^54, 55^. The reason for the apparent reinvention of methylation-based silencing in vertebrates is unclear. Comparison of TE dynamics in the cells of elephant shark and basal chordates offers the opportunity to determine whether the need for additional control mechanisms was a primary driver for genome-wide hypermethylation in jawed vertebrates.

We note that in addition to substantial physiological changes, the emergence of jawed vertebrates was accompanied by major innovations in gene regulatory networks, notably non-coding RNA elements ^49^. These advances may have facilitated, or conversely been enabled by, the development of a complex methylation system during the same time period. The role of the whole-genome duplications that occurred in vertebrate progenitors ^56^ in the acquisition of components that act downstream of the methylation signal, or as a stimulus for new mechanisms of regulating gene dosage, also merits further investigation. 

Methylation of elements that modulate gene expression forms an epigenetic memory that plays an important role in defining and stabilising cell identity in higher vertebrates ^5, 17– 21^. The reprogramming of this specification in the germline to regenerate full developmental competence after fertilisation, and the pathways employed to achieve this – such as active demethylation by ten-eleven-translocase (TET) enzymes, vary considerably across vertebrates ^57^. Examination of these phenomena in the elephant shark will provide insight into the evolutionary history of epigenetic control in the life cycle and its consequences for vertebrate development.

Our findings provide fresh perspective on an important epigenetic modification. The elephant shark methylome delineates the evolutionary extent of the complex methylation system found in higher vertebrates, and sets the scene for comparative studies that will address longstanding questions about the primary purpose of this system and how these functions evolved from the mosaic pattern of invertebrates. It will be particularly pertinent to understand the development of the mechanism that links TSS methylation to transcriptional repression. Epigenetic studies in the elephant shark also open promising avenues to explore the ways in which methylation is put to use during development and the specification of cell fate, and the conservation of these strategies amongst vertebrates.

## Data availability

The data referenced by this article are under copyright with the following copyright statement: Copyright: © 2017 Peat JR et al.

Data associated with the article are available under the terms of the Creative Commons Zero "No rights reserved" data waiver (CC0 1.0 Public domain dedication). 

All raw WGBS-seq data (including low-coverage WGBS-seq data), as well as mapped CG call files for male and female liver deep-sequencing, are deposited in the GEO database under accession number GSE96683.

Source for published WGBS-seq datasets:

Mapped CG methylated calls for mouse liver ^5^ were downloaded from GEO, accession GSE42836, sample GSM1051157.

Raw sequencing data for zebrafish muscle ^8^ were downloaded from SRA, study SRP020008, run SRR800081.

Raw sequencing data for sea squirt muscle ^14^ were downloaded from GEO, accession GSE19824, sample GSM497251.

Source for published RNA-seq datasets:

Raw sequencing data for elephant shark liver ^49^ were downloaded from SRA, SRA study SRP013772, run SRR513760.

Raw sequencing data for mouse liver (ENCODE Consortium ^50, 51^) were downloaded from GEO, accession GSE78583, sample GSM2072415.

Raw sequencing data for zebrafish muscle ^8^ were downloaded from SRA, study SRP020008, run SRR800045.

Raw sequencing data for sea squirt muscle ^14^ were downloaded from GEO, accession GSE19824, sample GSM497252;

## Supplementary material

**Table S1: Whole-genome bisulfite sequencing of elephant shark somatic tissues.**

The table lists the number of cytosine calls at either symmetric CG dinucleotides ('CG') or in other sequence contexts ('non-CG'), mapped against the elephant shark 6.1.3 genome assembly or mitochondrial DNA. Details of bioinformatic processing are provided in the methods section. For deep-sequenced samples, the number of calls following deduplication are given. The frequency of non-CG methylation indicates the maximum rate of non-conversion during the bisulfite treatment step; by this measure, all libraries had a bisulfite conversion efficiency of at least 98.9%.

.

**Figure S1: Bootstrap sampling to determine margin of error in low-coverage WGBS-seq.**

Empirical prediction of the margin of error (99% confidence interval) associated with low coverage WGBS-seq, as calculated by bootstrap sampling of the deep-sequenced male elephant shark liver dataset. Details of the sampling approach are provided in the methods section. An asymptotic model with the equation $y=2.208/\sqrt{x}$ was used to fit a curve to the data. At our minimum sequencing depth of 15,000 CG calls, bootstrap sampling predicts a margin of error of approximately ±1.8 methylation percentage points.
