# Supplementary material for: The elephant shark methylome reveals conservation of epigenetic regulation across jawed vertebrates
Source: F1000Res. 2017 Apr 20;6:526. [Version 1] doi: 10.12688/f1000research.11281.1 (PMC5437953; doi:10.12688/f1000research.11281.1)
Supplement: Peer review history

# Peer review: Referee response for version 1

**DOI:** 10.5256/f1000research.12170.r22066

**Published:** 2017-05-18

**Authors:** Arthur Georges, Changwei Shao

**Affiliations:** 1 Institute for Applied Ecology, University of Canberra, Canberra, ACT, Australia; 2 Yellow Sea Fisheries Research Institute, Chinese Academy of Fishery Sciences, Qingdao, China

In this paper, the authors report use of whole-genome bisulfite sequencing to generate the first genome-wide methylation profiles for a cartilaginous fish, the elephant shark *Callorhinchus milii*. The results are preceded by an excellent introduction to the topic, pointing to the pertinent recent literature. The work is significant because the cartilaginous fish arise form a lineage that branches early from the vertebrate phylogeny and their methylation profiles have not before been examined. The authors are able to compare this methylation profile with that of other vertebrates – zebrafish, mouse and sea squirt -- to show that the cartilaginous fishes exhibit a methylation profile that is characteristic of vertebrates generally, and in contrast to the pattern shown by invertebrates, including the chordate sea squirt. They were also able to report an inverse relationship between TSS methylation and gene expression in the elephant shark, supporting the presence of the repressive regulatory architecture shared by other vertebrates so far examined. This study narrows considerably the evolutionary window in which the widespread methylation pattern characteristic of vertebrates evolved. They demonstrate conservation of a complex methylation system across jawed vertebrates separated by 465 million years of evolution.

 The interpretation of the results is sound for the most part, and gives sufficient evidence to support their conclusion of conservation of methylation across jawed vertebrate, which fills the gap in our knowledge on the methylation system of vertebrate compared with invertebrate.

 We have concerns about the detail presented on the methylation data to support the conclusions. Information on the basic methylation data is an omission that needs to be rectified, and the reasons to focusing solely on CpG sites for further analyses. What quantity of methylome data was produced, what was the average depth per strand for each sample, and what was the mapping ratio?  What was the density of methylated genomic cytosines and how did the detailed distribution pattern of mCs differ with context?

 The most interesting point of the paper is that the elephant shark resembled the methylation pattern of other vertebrate lineages based on the characterization of genomic methylation.  DNA methylation is catalyzed by the three DNA methyltransferases (DNMT1, 2 and 3). Is it possible to also add a comparative analysis of DNMT to see if the cartilaginous fishes also resemble other vertebrate lineages (multiple copy) in comparison with invertebrates (single copy), including the non-vertebrate chordates?

 The authors interpret similarity of male and female methylation to indicate that, unlike some bony fish species, the uncharacterised sex-determination mechanism in the elephant shark is not associated with large differences in global methylation. We think the authors mean that the outcome of sexual differentiation in the elephant shark does not yield, or is not governed by, large differences in global methylation. Specifically, the reference to sex related differences in methylation in the bony fish species is to work that demonstrates a difference between ovary and testes. As such it refers to differences in tissue specific patterns of methylation, not necessarily related to sex determination itself.

 There is a semantic point, and the authors may wish to word it out of the manuscript. It centres on the use of basal taxa and higher vertebrates. The elephant shark studied is extant and so has had as much time to evolve and diverge from the common ancestor as we have. We suggest replacing references to the elephant shark as basal by "the elephant shark has arisen from an early branch of the vertebrate phylogeny". The logic on the implications of the results remains unchanged. Also consider wording out "higher vertebrates" with "other vertebrate lineages".

 Apart from those points, the authors might like to address some repetition of points made in the manuscript with a view to removing them and tightening up the manuscript. There appears to be an error in the caption of Figure 5 which states that Quintile 4 is omitted for clarity, but it appears to be included.

 We really liked this paper. it is an excellent contribution to our understanding of the evolution of the distinctive global methylation pattern of vertebrates. The work in couched in an introduction to the topic that provides the reader with a good introduction to the topic of global methylation patterns, by which to place the work in context.

We have read this submission. We believe that we have an appropriate level of expertise to confirm that it is of an acceptable scientific standard.

# Peer review: Referee response for version 1

**DOI:** 10.5256/f1000research.12170.r22183

**Published:** 2017-05-11

**Authors:** Matthew M. Hindle

**Affiliations:** 1 The Roslin Institute, University of Edinburgh, Edinburgh, UK

Peat *et al.* reveal the first methylome of a cartilaginous fish. A valuable contribution to comparative epigenetics that sheds light on the emergence of DNA methylation as a regulatory mechanism in vertebrate genomes. The lack of any published methylomes between Sea Squirts and Zebrafish emphasises just how important this paper is for understanding the evolution of epigenetics as a mechanism for transcript regulation.

 The paper is a great read and an excellent example of how to present genome-wide methylation data in an interesting and compelling format. The main claim that the elephant shark genome has a “higher vertebrate” like methylation profile is convincingly made in Figure 3. I am also persuaded by the higher vertebrate like correlation of CpG methylation and transposable elements (Fig 4B) and TSS (Fig 5 A-D). It is particularly encouraging that they managed to show such a convincing correlation in a non-model species, where genomic annotation is often inaccurate and incomplete. It is tempting to infer that the differences between the elephant shark methylome and those of higher vertebrates indicate an evolutionary intermediate/transitionary stage in the functional importance of TSS methylation to repress transcription (authors indicate there may be "less absolute repression than in higher vertebrates"). However, the authors appear to be very cautious in limiting their claims and acknowledge that the reduced amplitude of the TSS methylation correlation in Fig4B compared to mouse and zebrafish could be due to TSS miss-annotation in a non-model genome. It will be interesting to see if improvements of TSS annotation with CAGE or similar data alter the differences in the TSS methylation ratio. Given the authors carefully qualify their claims on the observed differences of elephant shark methylomes to higher vertebrates, it would be unreasonable to request that they improve on the reference annotation. Similarly, the transcript correlation differences between elephant shark and higher vertebrates in Figure 5 E-F could be the result of incomplete or erroneous transcript models. However, it is very interesting that compared to higher vertebrates there appears to be underrepresentation of CpG methylation for gene bodies in low-level expressed genes and for TSS an slight overrepresentation of CpG methylation in highly expressed genes. Again it is a tantalising finding but difficult to draw conclusions because of the annotation quality differences and the differences in the RNASeq datasets. 

 It is a shame the WGBS Sea squirt and Zebrafish data is muscle rather than liver (Table 1) but the authors made use of what was available.

 I use a similar Babraham pipeline for BS data and all the methods appear to be appropriate.

I have read this submission. I believe that I have an appropriate level of expertise to confirm that it is of an acceptable scientific standard.
